# Stock and cryptocurrency trading and problem gambling behavior during early phases of the COVID-19 pandemic: a narrative literature review

**DOI:** 10.3389/fpsyg.2025.1585094

**Published:** 2025-07-30

**Authors:** Natalie Leong Wei Lyn, Hui Yu Yeo, Claudia Choong Startup, John Ming Yan Koh, Vinh-Long Tran-Chi, Cyrus Su Hui Ho, Tji Tjian Chee

**Affiliations:** ^1^National University Hospital, Singapore, Singapore; ^2^Changi General Hospital, Singapore, Singapore; ^3^Faculty of Psychology, Ho Chi Minh City University of Education, Ho Chi Minh City, Vietnam; ^4^Department of Psychological Medicine, National University of Singapore, Singapore, Singapore; ^5^Department of Psychological Medicine, National University Hospital, Singapore, Singapore

**Keywords:** trading, gambling, addictive behavior, impulse control disorders, cryptocurrency

## Abstract

**Background:**

The Coronavirus Disease of 2019 (COVID-19) resulted in a global shift in gambling and trading behaviors. At present, a gap exists in understanding the relationship between excessive trading behavior and problem gambling, especially during the COVID-19 period. This narrative review analyzed (1) the changes in trading and gambling activity during the COVID-19 pandemic, (2) whether the pattern of trading activity resembles problem gambling, and (3) whether excessive trading and problem gambling share similar consequences.

**Methods:**

We searched databases such as Medline, PsychINFO, Scopus, and Google Scholar using relevant keywords, and included 60 reports for narrative synthesis.

**Results:**

During the COVID-19 pandemic, there were major changes to trading behavior, possibly due to market sentiments and psychology, personal financial needs, social media influence, and the behavior of other investors. The progression of the pandemic led to an increase in brokerage account openings and an increase in trading activities among existing investors, likely due to the development of digital trading platforms that enhanced accessibility for technology-adept investors. There was also a shift from gambling at physical destinations to online gambling, with an increase in frequency and spending among individuals who continued gambling. Feelings of boredom, stress, and the need for relaxation may motivate people to engage in gambling.

**Conclusion:**

Individuals who engaged in excessive trading and problem gambling shared similar traits and may thus face similar psychiatric consequences. The findings indicate that we can apply the diagnostic criteria for pathological gambling and gambling disorders to excessive trading, given that many of these individuals meet the criteria for an addictive disorder.

## 1 Introduction

While stock trading is largely influenced by macroeconomic factors, regulatory policies, and fundamental analysis, cryptocurrency trading is driven more by market sentiment, speculation, and technological trends, making it resemble gambling in its volatility and risk-taking behavior, particularly when traders engage without proper knowledge or strategy (Kou et al., 2024).

### 1.1 Gambling behaviors

Gambling, defined as the activity of wagering something of a particular value with the possibility of obtaining something of a higher value (Potenza et al., 2002), is commonplace in many parts of the world. The prevalence of gambling is high at 26% of the world population, amounting to approximately 1.6 billion people (Casino.org, 2021). Different forms of gambling exist, broadly classified into traditional gambling and online gambling. Traditional gambling includes casino games such as roulette, lottery, blackjack, and poker, as well as racing and sporting events (Potenza et al., 2002). With the constantly advancing technological landscape, it is unsurprising that traditional gambling has given way to a new form of gambling online, which includes any form of gambling carried out on the Internet. The global online gambling market was valued at approximately 59 billion U. S. dollars in 2019 and grew to an estimated 86 billion U. S. dollars by 2024, with projections suggesting it will reach around 120 billion U. S. dollars by 2029 (Statista, 2024). The rise in online gambling can be attributed to various reasons such as increased accessibility (Gainsbury et al., 2012; Griffiths and Barnes, 2008; Kim et al., 2017) and anonymity and privacy, allowing participants to gamble in the comfort and safety of their homes (Corney and Davis, 2010; Gainsbury et al., 2012).

### 1.2 Trading behaviors

Trading refers to “the activity of buying and selling financial instruments such as stocks, bonds, futures, commodities, and currencies” (Guglielmo et al., 2016). This umbrella term includes a range of behaviors from investing in longer-term holdings to day-trading, which is the act of trading stocks within the same 24-h period, with decisions made on the subtle variations in valuation in order to make a quick profit (Arthur and Delfabbro, 2017). Due to the large range of financial instruments available in trading, this review will focus on the buying and selling of Stocks and Cryptocurrencies. Whilst we acknowledge that the terms trading and investing are two separate entities, for the purpose of this paper, the two terms will be used interchangeably to denote the buying and selling of financial instruments, as a careful delineation between trading and investing behaviors was unlikely due to the ever-changing mindsets of individual participants in the markets.

### 1.3 Problem gambling and excessive trading

Gambling behaviors, whilst often perceived as a harmless and entertaining activity, can evolve into a habit that risks becoming a public health threat (John et al., 2020; Shaffer and Korn, 2002; The Lancet, 2017). Such negative consequences include increased risk of psychiatric disorders such as depression and anxiety, financial issues, as well as psychosocial issues (Awaworyi Churchill and Farrell, 2018; Golin, 2001; Griffiths and Barnes, 2008; Mills and Nower, 2019; Reith, 2006). Problem gambling behaviors had been clinically defined to provide better assessment and treatment for this addictive and compulsive behavior (Blaszczynski and Nower, 2010; Lorains et al., 2011; Gainsbury, 2015). In the Diagnostic and Statistical Manual of Mental Disorders (DSM-IV), Pathological Gambling, classified under the group of Impulse-Control Disorders Not Classified Elsewhere (American Psychiatric Association, 2000), was described as a “persistent and recurrent maladaptive gambling behavior.” Subsequently, it was renamed Gambling Disorder and classified under Substance-Related and Addictive Disorders in the DSM-V (American Psychiatric Association, 2013). Additional criteria of having to suffer “significant impairment or distress,” with certain behaviors demonstrated within the timeframe of 1 year, were added to the diagnostic criteria (American Psychiatric Association, 2013). Similarly for trading, motivation to engage in such behavior goes beyond the possibility of financial gains, with entertainment gaining traction as one of the major reasons for trading (Dorn et al., 2008). Some investors see trading as both a hobby and a form of gambling for entertainment (Dorn and Sengmueller, 2009). Excessive trading behaviors, however, have been viewed by the scientific community to be a form of addiction (Grall-Bronnec et al., 2017; Guglielmo et al., 2016) similar to gambling disorders (Grall-Bronnec et al., 2017; Guglielmo et al., 2016; Håkansson et al., 2021). Negative impacts such as depression, anxiety, as well as negative sequelae on financial and psychosocial areas, have been found to be associated with both excessive trading and gambling (Dixon et al., 2018; Grall-Bronnec et al., 2017; Guglielmo et al., 2016; Mills and Nower, 2019). Thus, the identification of high-risk individuals for these activities, and the extent to which they overlap, is essential and urgent.

The narrative review approach employed in this manuscript is employed to synthesize the most recent discoveries regarding behavioral changes in trading and wagering activities during the COVID-19 pandemic. This study adopts a narrative review approach, which allows for a broader synthesis of existing literature across multiple disciplines, including finance, psychology, and behavioral sciences. Unlike a systematic review, which is best suited for well-defined research questions with homogeneous study designs, a narrative review provides the flexibility to integrate diverse theoretical perspectives and empirical findings that may not be directly comparable. This approach enables a more comprehensive exploration of the economic and psychological influences on problematic trading and gambling behaviors while identifying critical gaps for future research.

### 1.4 Research aims

The COVID-19 epidemic has resulted in significant alterations in trading and gambling practices, although the existing literature lacks discourse on the correlation between excessive trading and problem gambling. This study specifically examines the impact of psychological, social, and economic variables stemming from the pandemic on impulsive and addictive behaviors, as physical trading venues and casinos were shuttered, resulting in a transition to online activities. This research aims to examine alterations in trading and gambling behaviors after the pandemic to ascertain if excessive trading signifies problem gambling and if conventional gambling diagnostics can be utilized to analyze trading behavior in this situation. These findings would facilitate a deeper understanding of the relationship between these behaviors in the context of a pandemic. Examples include social distancing measures and limits to public gatherings and “locking-down” of certain areas (Wilder-Smith and Freedman, 2020), an increase in the use of online gambling and trading platforms (Jenkinson et al., 2020), as well as experiences of social isolation, increased levels of tension, separation anxiety, and boredom (Brooks et al., 2020).

A notable gap exists in the understanding of associations between excessive, high-risk trading behavior and problem gambling during the COVID-19 period. While studies of this phenomenon in specific countries have already been conducted (Håkansson et al., 2021; John et al., 2020; Oksanen et al., 2022a; Oksanen et al., 2022b), there is a lack of global understanding of the associations between excessive trading and problem gambling across the world, which could be potentiated with the effects from the pandemic. Hence, this narrative review seeks to address the questions: To what extent do the characteristics of problematic trading behavior (stock and cryptocurrency) align with those of problem gambling, and can excessive trading be conceptualized within the framework of gambling disorder?

## 2 Methods

**Figure 1:**
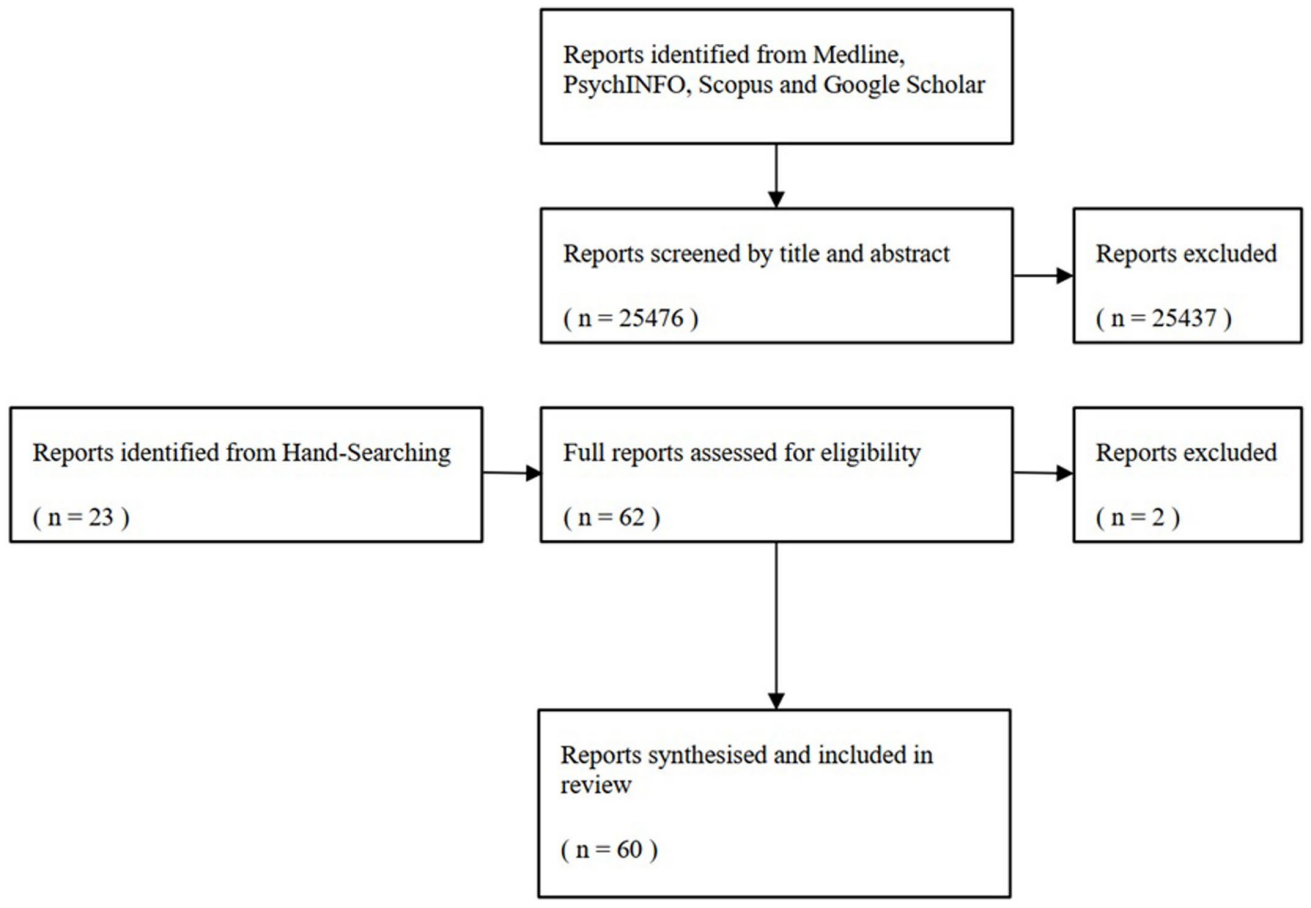
PRISMA flow diagram outlining the process of study selection and inclusion.

To carry out this narrative review, relevant databases including Medline, PsychINFO, and Scopus, as well as the first 200 records from Google Scholar as gray literature, were searched alongside hand-searching using combinations of search terms, such as Coronavirus, Covid, pandemic, trading, stocks, shares, cryptocurrency, investment, brokerage, problem gambling, addiction, excessive, behavior. The key papers and their findings were reviewed and discussed (Figure 1).

The inclusion criteria required that the studies (a) concentrate on trading and gambling behaviors associated with the COVID-19 pandemic, (b) utilize empirical data or a systematic review, and (c) be published in peer-reviewed publications. The exclusion criteria excluded literature that lacked an empirical foundation, studies that were not specifically focused on behavior changes related to the pandemic, and publications that were inaccessible in English.

## 3 Results

### 3.1 Effects of the COVID-19 pandemic on trading

The COVID-19 pandemic caused the fastest stock market crash in history. As the virus became more widespread, most stock markets across the globe experienced high sell-offs, causing them to become bearish. Lockdowns announced during the pandemic even caused trading halts on multiple occasions, as prices plunged more than 7% during the day and 5% at night (Bates, 2020). It is, therefore, unsurprising that the pandemic greatly affected investors’ sentiments and psychology (Naseem et al., 2021). Consequently, this has impacted trading activity across the globe in both quantitative and qualitative aspects. The included papers on the effects of COVID-19 on trading were summarized in Table 1.

**Table 1:** Summary of articles on COVID-19 and trading.

Authors	Origin	Sample	Purpose	Source	Findings
Abdeldayem and Al Dulaimi (2020)	Egypt	Part 1: NA Part 2: 318 investors in the Gulf Cooperation Council (GCC) stock market	Examined the causal association between expectations of pandemic risk and herding behavior.	Proceedings of Rijeka Faculty of Economics: Journal of Economics and Business	Expectations of pandemic risk have a significant positive impact on herding behavior in the GCC stock market.
Ahmad et al. (2020)	China	230 subjects (130 orphans, 100 non-orphans)	Examined risky investment behaviors made by orphans during the COVID-19 pandemic.	Psychology Research Behavioral Management (Journal)	Orphan investors made risky investment choices, and this behavior persisted even during a pandemic.
Bates (2020)	USA	N. A.	Summarized the effects of the COVID-19 pandemic on investor behavior.	Bachelor’s Thesis	During the COVID-19 pandemic, biases within behavioral finance, such as overtrading and overconfidence, were observed.
Bharti and Kumar (2022)	India	S&P CNX Nifty Index and its 50 constituent companies	Investigated the behavioral bias of market-wide herding in the Indian equity market during the spread of COVID-19 pandemic.	Millennial Asia: An International Journal of Asian Studies	Market volatility during the COVID-19 pandemic promotes significant herding behavior in the Indian equity market. The government’s control measures successfully reduced this behavior.
Bu et al. (2020)	China (Wuhan)	225 subjects, mostly graduate students	Examined how risk tolerance evolved during a worldwide health-crisis.	Sustainable Architecture for Finance in Europe	Greater exposure to COVID-19 caused less belief in luck and less sense of control; this led to more pessimistic views of the economy. Risk taking was affected by past experiences, and changes in risk taking were more affected by time-varying beliefs and optimism than general risk appetite.
Chang et al. (2020)	Taiwan	Renewable and fossil fuel energy markets in the USA, Europe, and Asia	Investigated herding in renewable energy, using daily closing prices in renewable and fossil fuel energy stock returns for March 24, 2000–May 29, 2020, which covers the Global Financial Crisis (GFC) (2007–2009), the coronavirus crises of SARS (2003), and the ongoing COVID-19 (2019–2020) pandemic.	Renewable and Sustainable Energy Reviews (Journal)	During SARS and COVID-19, herding was more prominent during extremely high oil returns. During COVID-19, herding was more prominent during extremely low oil returns. After the global financial crisis, investors were more sensitive to asset losses, causing them to be more likely to display herding in the stock market.
Chiah and Zhong (2020)	Australia	37 international equity markets	Examined the impact of COVID-19 on trading volume in stock markets around the world.	Finance Research Letters (Journal)	There was a large spike in trading volume in the 37 international equity markets studied. Investors trade more readily in countries that are wealthier, have stronger protection of legal rights, have better governance systems and have greater gambling opportunities.
Chin (2021)	Malaysia	271 investors	Examined the underlying psychological and sociological factors that drive excess trading in the Malaysian stock market during the COVID-19 pandemic.	Asian Journal of Business and Accounting	Investors with certain personality traits (higher neuroticism, lower extraversion, higher openness, higher agreeableness, lower conscientiousness) had higher trading frequency both pre and during COVID-19. Investors with certain demographics (younger age, male gender, greater household income, greater years of investment experience, being a full-time investor) also had higher trading frequency both pre and during COVID-19.
Dhall and Singh (2020)	India	N. A.	Examined the herding behavior at the industry level from national stock exchange (NSE).	Millennial Asia: An International Journal of Asian Studies	The COVID-19 Pandemic promoted herding behavior at the industry level, in India.
Espinosa-Méndez and Arias (2021a)	Chile	Australian stock market firm samples	Investigated if the COVID-19 pandemic has an effect on herding behavior in the Australian stock market.	Applied Economics Letters (Journal)	Herding behavior was observed in Australia during COVID-19. Investors initially abstained from investing during a health crisis.
Espinosa-Méndez and Arias (2021b)	Chile	Stock exchange samples of France (Paris), Germany (Frankfurt), Italy (Milan), UK (London), Spain (Madrid)	Investigated whether COVID-19 pandemic had an effect on herding behavior in Europe.	Finance Research Letters (Journal)	COVID-19 increased herding behavior in the capital markets of Europe. Fear and uncertainty during the pandemic might have caused less informed investors to discard their own beliefs and to follow more informed investors instead.
Fang et al. (2021)	China and Taiwan	Stock market samples of Russia, Poland, the Czech Republic, Hungary, Croatia, and Slovenia	Examined how and whether the COVID-19 pandemic affects herding behavior in the Eastern European stock markets.	Frontiers in Public Health (Journal)	Herding behavior in Eastern European markets were more prominent during COVID-19.
Gurav and Kotrappa (2020)	USA	N. A.	Analyzed investor’s sentiments considering US presidential elections and effects of COVID-19 as an explicit fluctuating factor affecting stock market performance	SSRG International Journal of Engineering Trends and Technology	Tweets regarding the pandemic and other major events (such as the US presidential elections) can be used to gauge investor sentiments and thus, the performance of the stock market via machine learning.
Himanshu et al. (2021)	India (Delhi and Mumbai)	184 investors	Analyzed the impact of COVID-19 on the portfolio allocation decisions of individual investors.	Journal of Public Affairs	During the financial crisis caused by COVID-19, most investors were moving toward a more conservative portfolio. However, some investors were risk-takers – they not only kept prior investments in stocks but also invested more funds in stocks. Some investors also chose not to change their existing portfolio.
Hong et al. (2020)	China	ChiNext Market	Investigated the presence and the asymmetric effects of investor herding in the ChiNext market over the period from October 30, 2009, to April 30, 2020	Romanian Journal of Economic Forecasting	Herding behavior was seen in this market, even after controlling for the effects of COVID-19. This behavior is more prominent during bearish periods, and in certain industries--including the manufacturing and IT sectors.
Khanthavit (2020)	Thailand	N. A.	Investigated the behavior of foreign investors in the Stock Exchange of Thailand in the time of COVID-19 as to whether trading is abnormal, what strategy is followed, whether herd behavior is present, and whether the actions destabilize the market.	Journal of Asian Finance, Economics and Business	Foreign traders’ abnormal trading volume is negative and significant during the COVID-19 period. Furthermore, foreign traders are not positive-feedback investors and they self-herd.
Kizys et al. (2021)	UK	72 stock market indices across the world	Studied if government response to the novel coronavirus COVID-19 pandemic can mitigate investor herding behavior in international stock markets.	International Review of Financial Analysis (Journal)	Herding behavior was observed in the first 3 months of 2020, and a more stringent government response mitigates such behavior.
Krokida et al. (2020)	Greece	EU and US sample markets	Studied the relationship between conventional and unconventional central bank monetary policy and herd behavior in equity markets.	Journal of Economic Behavior & Organization	The channel through which monetary policy may affect herding behavior is economic expectations and investor sentiment.
Luu and Luong (2020)	Vietnam and Taiwan	N. A.	Identified the difference between the herding behavior of emerging market and frontier market during pandemics.	Journal of Asian Finance, Economics and Business	Up to 12 industries demonstrated herding behavior in Vietnam, while only 5 of 17 industries demonstrated herding behavior in Taiwan. Different industries respond differently during influenza pandemics.
Mand et al. (2023)	Malaysia	Sample for conventional stocks, Islamic stocks and sample for the whole market	Investigated whether market conditions have an effect on investors’ propensity to herd in an emerging economy’s stock market	PSU Research Review (Journal)	Herding behavior existed among Shariah-compliant during up and down markets
Mnif et al. (2020)	France	Cryptocurrency Markets (Bitcoin, Ethereum, Ripple, Litecoin, Binance)	Investigated the herding biases by quantifying the self-similarity intensity of cryptocurrency returns during the COVID-19 pandemic.	Finance Research Letters (Journal)	Herding behavior was observed in the 5 top cryptocurrency markets: Bitcoin, Ethereum, Ripple, Litecoin, Binance. However, such behavior was reduced after COVID-19 and all of these cryptocurrencies became more efficient after the pandemic.
Mnif and Jarboui (2021)	France	Bitcoin market	Analyzed the Bitcoin dynamics and the investor response by focusing on herd biases.	Review of Behavioral Finance (Journal)	Bitcoin was more efficient after the pandemic, showing that the pandemic reduced herding bias.
Naseem et al. (2021)	China, Japan, and the United States	N. A.	Analyzed investor psychology and stock market behavior during COVID-19.	Frontier Psychology (Journal)	The pandemic had a negative impact on investor sentiments, causing a downward trend in the 3 stock markets analyzed.
Ortmann et al. (2020)	UK	N. A.	Examined how retail investors responded to the outbreak of COVID-19.	Finance Research Letters (Journal)	Trading intensity increased by 13.9% as the number of COVID-19 cases doubled. This behavior was more prominent among investors who are male and older.
Gügercin and Richter (2021)	Germany	108 participants	Investigated to what extent overconfidence influences the trading volume during the COVID-19 pandemic.	Master’s Thesis	Younger and inexperienced traders entered the market in 2020. Retail investors with more than 2 years of trading experience significantly increased their trading volume during COVID-19. During this pandemic, traders who reported that they had above average trading skills traded significantly more.
Riyazahmed (2021)	India	753 investors	Analyzed the impact of investor motives and awareness on investor preferences.	Investment Management and Financial Innovations (Journal)	Following the COVID-19 outbreak, there was an increase in investment in shares, mutual funds, and life insurance in India. Furthermore, more young Indians were showing interest in high risky investments. Investors’ preferences were affected by their personal traits.
Valle-Cruz et al. (2022)	Mexico	N. A.	Analyzed if the polarity generated by Twitter posts influence the behavior of financial indices during pandemics.	Cognitive Computation (Journal)	Twitter posts influenced financial indices during both pandemics, but the effect was more significant during the COVID-19 pandemic. The drop in stock prices during the COVID-19 era was more dramatic than the H1N1 period as there were more speculation, rumors, and negative news.
Wen et al. (2022)	Hong Kong	N. A.	Conducted a systematic mechanism for herding detection in the Hong Kong stock market.	Journal of Chinese Economic and Business Studies	Herding behavior was observed in Hong Kong during the pre-Covid period (likely due to the social chaos and release of new housing policy), but not during the COVID-19 period.
Wu et al. (2020)	China	Shenzhen A share market and Shanghai A share market	Investigated herding behavior in the Chinese stock markets during the COVID-19 pandemic.	Emerging Markets Finance and Trade (Journal)	Herding behavior was significantly lower during COVID-19 period. Herding behavior was more significant during upside market movements, lower market trading volumes, and lower market volatility during COVID-19.
Yarovaya et al. (2021)	UK	Cryptocurrency Markets traded in USD, EUR, JPY, KRW	Analyzed herding in cryptocurrency markets in the time of the COVID-19 pandemic.	Journal of International Financial Markets	Herding behavior depended on up- and down-market days but was not more prominent during COVID-19.
Yuan (2021)	China	N. A.	Examined the existence of herding effect in Chinese A share main board market using both market-level and industry-level data by testing the non-linear relationship between cross-sectional absolute deviation of returns and market returns.	2021 7th International Conference on E-Business and Applications	Investors in China’s A share market exhibit herding behavior, and this was exacerbated during the COVID-19 outbreak. This behavior was also more prominent when there was a negative market rate of return. Different industries were affected to different degrees.

#### 3.1.1 Effects on online trading

During the COVID-19 pandemic, more brokerage accounts were opened worldwide—including the USA, Germany, and Singapore (Aw, 2020; Ortmann et al., 2020). It was further observed that among the new brokerage accounts, there were more younger and inexperienced traders who entered the market in 2020 than in previous years (Chiah and Zhong, 2020; Fitzgerald, 2020; Gügercin and Richter, 2021). These individuals were more likely to be overconfident than experienced traders, fueling more risky trading behaviors (Gügercin and Richter, 2021).

The increasing trend of new brokerage accounts was aided by the development of digital trading platforms, which allowed young, technology-adept investors to access the stock markets more readily (Aw, 2020; Tang and Mahmud, 2021). Furthermore, such platforms had very low commission fees compared to traditional brokerages, making trading more accessible to the younger generation, including students. In the USA, examples of such platforms include Robinhood, TD Ameritrade, Charles Schwab, and Etrade; in Asia, Tiger Brokers, Moo Moo, POEMS, and Saxo Markets (Blystone, 2022; Reinkensmeyer and McKhann, 2022; Teo, 2022).

As online trading platforms gained popularity among the younger generations of inexperienced traders, experts were reportedly concerned about this trend (Tang and Mahmud, 2021), as younger investors were more likely to indulge in riskier trading behaviors (Ahmad et al., 2020; Riyazahmed, 2021).

#### 3.1.2 Effects on trading volume

As with the opening of more brokerage accounts worldwide, trading volume and intensity also increased during the COVID-19 pandemic. In the UK, as the number of COVID-19 cases doubled, the average weekly trading intensity increased by 13.9%, largely fueled by increased stock and index trading (Ortmann et al., 2020). Both new and experienced investors also added more funds into their trading accounts as the number of COVID-19 cases increased. A similar trend was observed in India, where there were greater investments in shares, mutual funds, and life insurance during the outbreak (Riyazahmed, 2021). This increase in trading volume was especially prominent in males, older investors, and traders who perceived themselves to be more skillful in trading (Gügercin and Richter, 2021; Ortmann et al., 2020).

#### 3.1.3 Risk factors of increased and excessive trading

Investor behavior and sentiments were affected by various internal and external factors. Internal factors that shaped investors’ behaviors include their demography, personality, past experiences in the stock market, and financial needs. On the other hand, external factors include traditional media platforms, social media, and the behavior of other investors. Given the combined effect of these internal and external risk factors on investor psychology, trading activity saw major changes as a result of the pandemic.

Possible risk factors for increased and even excessive trading behavior during the COVID-19 period included demographics, personality, psychological factors, as well as the influence of prior trading experiences and the behavior of other investors (evidenced in herding behavior).

#### 3.1.4 Demographic risk factors

Studies on how investor demographics affect trading behavior with regards to the frequency of trade and risk tolerance have been conducted prior to the pandemic. It was observed that people of younger age, male gender, greater household income, and greater years of investment experience tended to invest more frequently, a pattern that was consistent even during the COVID-19 pandemic (Chin, 2021). Regarding risk tolerance, many authors expected the pandemic to cause a decrease in risk tolerance for investments due to fear, uncertainty, pessimism, and feelings of lack of control. Such sentiments appear to be shared in India (Himanshu et al., 2021) and China (Bäckman et al., 2020), where investors preferred more conservative portfolios. However, there were exceptions with certain groups of investors within the population (Himanshu et al., 2021; Riyazahmed, 2021). Demographic features of investors who demonstrated higher risk tolerance during the pandemic included males, younger investors, and investors who earned lower incomes (Ahmad et al., 2020; Riyazahmed, 2021). Interestingly, orphans were also noted to be greater risk-takers during the pandemic (Ahmad et al., 2020). It was hypothesized that orphans are used to taking risks from childhood due to the lack of parental figures, causing them to make riskier decisions in investments.

#### 3.1.5 Personality risk factors

Apart from demographics, personality traits also played a role in affecting investment volume and choices. Both before and during the pandemic, it was observed that investors with higher neuroticism, lower extraversion, higher openness, higher agreeableness, and lower conscientiousness traded more compared to their counterparts (Chin, 2021). In comparison, investors who were more extraverted and conscientious were less likely to make changes to their trading behavior during the pandemic, as they were found to be more emotionally stable, judicious, and cautious when making investment decisions (Chin, 2021).

#### 3.1.6 Influence on prior trading experiences

Personal experiences shape perspectives and affect decision-making, even in the aspect of investments. During the COVID-19 pandemic, two types of experiences were found to affect investors’ behavior. First, prior experience from gains and losses in the stock market affected investors’ risk-taking and herding behavior during the pandemic. Prior gains in the market led to riskier investing, while prior losses led to reduced risk-taking in investors from Delhi and Mumbai (Himanshu et al., 2021). Second, personal experiences of COVID-19 exposure and health risks affected investors’ risk tolerance. In Wuhan, people who had higher risk of being exposed to and infected by the coronavirus reflected greater fear regarding the pandemic, and this group of people demonstrated lower risk tolerance in investments (Bäckman et al., 2020).

#### 3.1.7 Influence of other investors (herding behavior)

Herding behavior refers to the act of following the trading decisions made by others (i.e., follow the crowd), rather than making an informed decision based on personal convictions, self-analysis, or research. This behavior has been described as problematic as it drives baseless and irrational market rallies, leading to fads and asset bubbles which might eventually “burst” (Krokida et al., 2020). For example, the concerted buying of the Gamestop stock (ticker symbol GME) by online communities was widely attributed to a Reddit sub-community known as the “Wall Street Bets” and certain influential figures (Verlaine and Banerji, 2021). As a result, the price movement of GameStop’s stock prices became very volatile, and rose by more than seven times in 2021 (Osipovich, 2021; Verlaine and Banerji, 2021), arguably beyond its fair valuation.

In the first half to late 2020, herding behavior was observed across various countries’ markets, including the Gulf Cooperation Council Countries (GCC) (Abdeldayem and Al Dulaimi, 2020), Oceania (Espinosa-Méndez and Arias, 2021a), Vietnam (Luu and Luong, 2020), India (Bharti and Kumar, 2022) and Europe (including France, Germany, Italy, the UK, Spain, Russia, Poland, Czech Republic, Hungary, Croatia, and Slovenia) (Espinosa-Méndez and Arias, 2021b; Fang et al., 2021; Kizys et al., 2021). Herding behavior was observed to be amplified by the pandemic due to heightened fear, uncertainty, and expectations of pandemic risk, especially among less informed agents (Bharti and Kumar, 2022; Dhall and Singh, 2020; Yuan, 2021). As a result, less informed agents abandoned their beliefs and followed others, leading to market inefficiencies.

Studies on herding behavior during the “early” phases of the pandemic found mixed outcomes (Chang et al., 2020). For instance, three studies observed increased herding behavior in China during the pandemic (Hong et al., 2020; Luu and Luong, 2020; Yuan, 2021), but one study found reduced herding behavior instead (Wu et al., 2020). In Hong Kong, mild herding was observed in three sectors, i.e., banking, real estate, and state-controlled before COVID-19, but this phenomenon was weakened across all 3 sectors during the pandemic (Wen et al., 2022).

Herding behavior was also observed to be asymmetric depending on the market trend and industry (Hong et al., 2020; Yuan, 2021), being more significant during market uptrends, in the manufacturing and IT sectors, and in large and small-sized portfolios compared to medium-sized portfolios (Hong et al., 2020; Yuan, 2021). This was seen in a study of the Shanghai A and Shenzhen A share markets, where herding behavior was found to be more significant during market uptrends (Wu et al., 2020). The reasons for increased herding behavior (Hong et al., 2020) were suggested as follows: (1) for market uptrends, investors tended to buy more stocks in anticipation of a further rise in prices, while during market downtrends, investors might hold on to their stocks as they expect their losses to be reversed, (2) manufacturing and IT sectors were high-tech sectors which dominated the market, (3) large portfolios as they are more featured in the media, making it easy for investors to follow market trends, whereas (4) smaller portfolios received less attention by the media, and the relative lack of information caused investors to follow market consensus rather than make their own decisions.

In contrast, studies have found reduced herding behavior in the cryptocurrency markets during the pandemic. Decreased herding was observed in the top five cryptocurrency markets, including Bitcoin, Ethereum, Ripple, Litecoin, and Binance (Mnif et al., 2020), as well as in the USD and Euro cryptocurrency markets despite the increased volatility in these markets (Yarovaya et al., 2021). This unexpected trend was hypothesized to be a result of the conventional expansionary and non-standard policies by governments (Yarovaya et al., 2021) and their effect on investors’ sentiments and expectations of the economy. With increased central bank credibility, investors could have been more confident with more positive sentiments in their decisions, leading to reduced herding behavior (Krokida et al., 2020).

### 3.2 Effects of the COVID-19 pandemic on gambling

During the COVID-19 pandemic, fewer people were participating in gambling activities in general. Results from a study in the United Kingdom (UK) showed an initial decrease in gambling frequency in the first month of lockdown. Some studies attributed this to decreased accessibility of gambling venues, such as with postponement or cancelations of sporting events and closures of gambling destinations (Sharman et al., 2022). The papers on COVID-19 and gambling were summarized in Table 2.

**Table 2:** Summary of articles on COVID-19 and gambling.

Authors	Origin	Sample	Purpose	Source	Findings
Barrault et al. (2017)	France	416 regular online poker players, recruited from the most active Internet poker-related forums in France	Assessed emotion regulation, anxiety and depression in a sample of regular poker players, and to compare the results of problems and non-problem gamblers.	Journal of Gambling Studies	Anxiety and depression were significantly higher among severe-problem gamblers than among the other groups. Both significantly predicted problem gambling.
Daglis (2022)	Greece	N. A.	Tested the effect of the COVID-19 pandemic on excessive gaming and gambling activities.	Journal of Economic Studies	A strong and positive long-run relationship exists between COVID-19 and gaming and online gambling companies’ stocks, unveiling a long-lasting and important effect. The small fluctuations are more persistent than the large ones, and a rich multifractal spectrum exists in this relationship.
Economou et al. (2019)	Greece	3,404 participants from the telephone survey and 2,400 participants from the patron survey	Investigated the influence of the recession on problem gambling and to identify its correlations, with heightened interest in socio-economic correlates.	Journal of Gambling Studies	Findings indicated that 2.4% of respondents met criteria for problem gambling. Male gender, minority status, living with family of origin, low educational level and low to zero income were found to constitute risk factors of the disorder. Moreover, having started gambling during the recession increased the odds of suffering from problem gambling.
el-Guebaly et al. (2006)	Canada	14,934 respondents	Compared gambling behaviors in a random sample of community residents with and without mental disorders identified by the Composite International Diagnostic Interview (CIDI).	Journal of Gambling Studies	5.8% of the respondents, who engaged in at least one type of gambling activity in the past 12 months in 2006, fell in the low severity gambling category, while 2.9% fell in the moderate/ high category. The risk of moderate/ high severity gambling was 1.7 times higher in persons with mood or anxiety disorder compared to persons with no selected disorder. For people with substance dependence or harmful alcohol use, the risk of moderate/ high severity gambling was 2.9 times higher. People with both disorders were 5 times more likely to be moderate/ high severity gamblers.
Håkansson (2020)	Sweden	2,016 participants	Examined whether self-reported gambling has increased during the pandemic, and the potential correlates of such a change.	International Journal of Environmental Research and Public Health	There was a 4% increase in overall gambling during the period 24 April to 3 May 2020. The proportion of individuals reporting increases in gambling was markedly higher for online casinos, online horse betting and online lottery compared to those who reported decreases in gambling. Overall, gambling increases were independently associated with gambling problems and alcohol consumption.
Hodgins and Stevens (2021)	Canada	Review of 17 articles and reports	Systematically identified and describe the survey data and findings to date examining the effect on individual gambling and gambling disorder	Current Opinion in Psychiatry (Journal)	Overall reduction in gambling frequency and expenditure was reported in all 17 studies, given the closure of land-based gambling venues, from March to May 2020. The proportion of participants in both the general population and the population that gambled who reported an increase in overall gambling was more variable and ranged from 4 to 14% in 4 studies providing this information. A greater number of studies estimated the increase in online gambling specifically. The most consistent correlates of the subgroup of people that increased gambling during the lockdown was increased problem gambling severity, younger age groups and being male. Impacts of the COVID-19 pandemic on gambling and problematic gambling are diverse – possibly causing a reduction in current or future problems in some, but also promoting increased problematic gambling in others.
Islam et al. (2020)	Bangladesh	13,525 individuals, aged 18–50 years old	Assessed problematic internet use among Bangladeshi youth and adults in Bangladesh and examined its correlation with lifestyle and online activities during the COVID-19 pandemic.	Addictive Behaviors Reports (Journal)	Problematic internet use was strongly associated with socio-demographic factors (being younger in age, having a bachelor degree level of education, being unmarried, being a member of a nuclear family, having middle-income socio-economic status, living in an urban area), lifestyle factors (being a cigarette smoker, being a heavier sleeper, being physically inactive, not engaging in household chores), and online behaviors of an individual (internet browsing hours, playing online games, social media purposes, recreational activities).
Olason et al. (2015)	Iceland	8,249 adults	Reported on the results of three national prevalence studies conducted before and after the economic collapse in Iceland.	Journal of Gambling Studies	There was an increase in the year 2015 gambling participation which extended across most gambling types. Prevalence of problematic gambling increased over the past year, but further examination revealed that this increase was most probably explained by an increase in card and internet gambling among young men. Moreover, those who experienced financial difficulties due to the economic recursion were 52% more likely to have bought a lottery ticket during the recession compared to those who were not affected financially.
Price (2022)	Canada (Ontario)	2005 gamblers, including a subsample of 1,081 online gamblers	Examined the emerging impact of COVID-19 on gambling during the first 6 weeks of emergency measures in Ontario, Canada.	International Journal of Mental Health Addiction	There was a significant likelihood of online gambling among those classified as high-risk gamblers, according to the Problem Gambling Severity Index and those with past experience of online gambling. Among high-risk online gamblers, the most predictive risk factors included moderate and severe anxiety and depression, reduced work hours, being influenced to gamble due to COVID-19, gambling under the influence of cannabis or alcohol, and risky gambling motives tied to mental health concerns, including gambling because it helped with nervousness and depression, chasing gambling losses and seeking to earn income.
Sharman et al. (2022)	UK	1,028 adults	Captured the immediate lockdown-enforced changes in gambling behavior.	International Journal of Mental Health Addiction	Gambling participation decreased between pre-lockdown and during lockdown. Both gambling frequency and weekly expenditure decreased during the first month of lockdown overall, but the most engaged gamblers did not show a change in gambling behavior, despite the decrease in opportunities and availability of gambling avenues. Individuals whose financial circumstances were negatively affected by lockdown were more likely to perceive an increase in gambling than those whose financial circumstances were not negatively affected.
Gambling Commission (2020)	UK	Around 2,000 adults through YouGov survey	Summarized the general consumer gambling trends in the UK during the COVID-19 pandemic.	The Gambling Commission (Government Organization)	Overall, fewer consumers were gambling. Between March and April 2020, there was a 5% decrease in active player accounts, driven substantially by real event betting. Data showed that a third of existing gamblers were trying new gambling activities for the first-time during lockdown. Engaged gamblers had increased either amount of time or money spent on at least one gambling activity.

#### 3.2.1 Effects on gambling volume

Among individuals who continued gambling during the COVID-19 period, there was an increase in frequency and spending on gambling activities. A study by the Gambling Commission in the UK found that despite fewer “active player accounts” between March and April 2020, more engaged gamblers reported increased expenditure and time spent on at least one gambling activity (Gambling Commission, 2020). Similarly, a subset of participants from a study in Sweden reported increases in gambling problems (Håkansson, 2020). This trend was also found in studies from Greece, Iceland, and the United States (US) during the 2008 Financial Crisis, where a significant rise in risky gambling behavior strongly associated with severe financial adversity was observed (Economou et al., 2019; Olason et al., 2015). While fewer people may be engaging in gambling behavior during the COVID-19 pandemic, more avid gamblers tended to spend more time and money on gambling during this period.

#### 3.2.2 Effects on online gambling

With COVID-19 movement restrictions in place, gambling activities shifted from physical destinations to online gambling. Many gambling venues, such as casinos, horse-racing tracks, bars and clubs with electronic gambling machines (EGMs), lottery retailers, betting shops, and poker rooms, faced closures in light of the lockdowns in March–April 2020 (Hodgins and Stevens, 2021). A systematic study in 2021 found an overall reduction in gambling frequency and expenditure, citing reasons such as the unavailability of live sports (22%) or canceled exclusive gambling on sports events (28%) (Hodgins and Stevens, 2021; Price, 2022). Simultaneously, as people spent more time at home, studies also noted an increase in online gambling by 11–20% (Hodgins and Stevens, 2021; Price, 2022). One study found that COVID-19 and the stocks of certain online gambling and gaming companies showed strong, persistent, and positive long-run relationships, suggesting that these activities were gaining popularity during the pandemic (Daglis, 2022).

#### 3.2.3 Risk factors of problem gambling during COVID-19

Studies have identified possible risk factors for problem gambling behavior during the COVID-19 period, such as demographics, psychological factors, and specific motivations such as boredom and stress relief.

#### 3.2.4 Demographic risk factors

A potential demographic risk factor for problematic online gambling behavior during the COVID-19 period was being of a younger age group. Studies suggest that being in the 18–25 years old age group was associated with increased problematic internet use, which includes gambling among other virtual activities such as gaming and social media use. Increased freedom from parental control and easier access to online applications were possible enabling factors for online gambling (Islam et al., 2020; Price, 2022). With more educational and occupational work shifting to the virtual landscape, young adults facing challenges adjusting to new lifestyles might have been more susceptible to online gambling during the pandemic (Islam et al., 2020).

#### 3.2.5 Psychological risk factors

Psychological factors such as loneliness, depression, and anxiety were postulated as risk factors for increased gambling to relieve stress during the pandemic (Islam et al., 2020). One study suggested that predictive factors of gambling included anxiety, depression, reduced working hours, and those with a previous history of online gambling. Those screened for moderate and severe forms of anxiety (25.7%) and depression (12.6%) were more likely to participate in online gambling during the earlier period of the pandemic (Price, 2022). Previous studies have also established an association between anxiety or depression and problematic gambling behaviors (Barrault et al., 2017; el-Guebaly et al., 2006).

#### 3.2.6 Motivational risk factors

Common motivations for those who participated in gambling during the COVID-19 pandemic include boredom, stress relief, and leisure. Among those with increased gambling behavior, boredom was identified as one of the more common motives, along with a need for relaxation (25%) and stress relief (15%) (Hodgins and Stevens, 2021). This was not surprising, given that many countries were going into lockdown during the pandemic, giving individuals more leisure time at home to participate in gambling activities.

### 3.3 Associations between pathological gambling and excessive trading

Having established the increased patterns of trading and gambling behaviors during the COVID-19 pandemic, the relationship, similarities, and differences between excessive trading and gambling disorders is explored in this section. Specifically, both excessive trading and problematic gambling share core behavioral features, including the tendency to chase losses, act on emotional impulses, and persist in the behavior despite adverse consequences (Grall-Bronnec et al., 2017; Marković et al., 2012). These behaviors are often reinforced by psychological mechanisms such as intermittent rewards, heightened excitement, and the illusion of control (Golin, 2001; Grall-Bronnec et al., 2017). Table 3 summarizes the papers exploring the relationships between gambling and trading.

**Table 3:** Summary of articles on trading and gambling.

Authors	Origin	Sample	Purpose	Source	Findings
Arthur and Delfabbro (2017)	Australia	9,508 adults	Established whether the findings of Arthur et al. (2015) can be replicated in an Australian context and when speculative stock market activity is defined in a narrower way (i.e., just day trading).	Journal of Gambling Studies	Majority of day traders were found to engage in traditional forms of gambling, with the level of participation and frequency of participation being significantly higher than that of the general adult population. Day traders preferred skill-based gambling formats as compared to chance-based formats. Prevalence of problem gambling among day traders was significantly higher than that of non-day traders.
Binde (2009)	Sweden	Reviewed 434 works on gambling	Presented a review of the literature on gambling in the social, economic, and cultural sciences, with a focus on people’s motives for participating in gambling and the factors that influence their degree of involvement.	Swedish National Institute of Public Health (Government Organization)	Risk factors for problem gambling include: (a) Direct risk factors: risk cognitions, risk practices (b) Indirect risk factors: social, emotional, biological predisposition, environmental conditions.
Dorn and Sengmueller (2009)	Germany	577 paper and 768 online survey responses and transaction records	Examined the hypothesis that entertainment motives drive trading for a sample of more than 1,000 clients at one of the top three discount brokers in Germany.	Management Science (Journal)	Investors who reported enjoying investing or gambling turned over their portfolio at twice the rate of their peers.
Gao and Lin (2015)	Taiwan	163 large jackpot lotteries above 500 million TWD	Investigated if individual investors treat trading as a fun and exciting gambling activity, implying substitution between this activity and alternative gambling opportunities	The Review of Financial Studies	Individual investors traded less on large jackpot days, suggesting a substitution effect between stock trading and lottery participation. Hypothesized reasons include buying and selling stocks being viewed as an alternative to fun and exciting gambling activities such as lotteries.
Golin (2001)	United States	249 participants from day trading and online trading groups	Examined the psychological factors underlying frequency of computer trading	PhD Thesis	Risk-taking and cognitive bias was positively correlated with tendency to gamble and with level of trading.
Grall-Bronnec et al. (2017)	France	8 excessive traders seeking treatment at the Problem Gambling Unit	Presented the phenomenological similarities between gambling disorders and a maladaptive and excessive way of trading.	Addictive Behaviors (Journal)	Excess trading showed similarity to addiction in behavior and the process of addiction (preoccupation, concealment, inability to control). The diagnostic criteria for gambling disorder could be applied to excessive trading, with trading and gambling having similarities in activity profile and structural characteristics. Sensation-seeking personality traits and illusion of control were evident in traders and gamblers.
Jadlow and Mowen (2010)	United States	1,158 participants	Investigated to what extent gamblers and stock investors share similar characteristics.	Journal of Behavioral Finance	Gamblers and investors share five trait characteristics (material needs, competitiveness, superstition, financial conservatism and numeracy).
Markiewicz and Weber (2013)	Poland	3,870 participants	Examined determinants of the trading pattern of day traders, who are commonly perceived to be risk takers and gamblers.	Journal of Behavioral Finance	Higher self-reported scores in DOSPERT (focusing on risk-accepting attitudes) are associated with greater number of trades, transaction costs and extent of day trading
Marković et al. (2012)	Croatia	111 participants	Determined whether the respondents who spend their time investing in the stock market meet the addiction criteria according to DSM-IV TR classification of psychiatric disorders.	Alcoholism (Journal)	Majority of respondents who trade met the DSM-IV criteria for addiction. Pathological gambling shared similarities with addiction in biological, behavioral, clinical and epidemiological aspects. Increased opportunities and ease of access to trading was positively associated with increased frequency of pathological gambling. Previous studies found similarities in personality profiles of traders and pathological gamblers.
McCormack and Griffiths (2013)	UK	Scoping study of 70 different internet gambling websites, 8 experts in the gambling research field, and unknown number of academic papers and “gray” literature.	Developed a comprehensive list of all the structural and situational characteristics of internet gambling and identified those which may be more problematic for internet gambling compared with offline gambling.	International Journal of Cyber Behavior, Psychology and Learning	38 structural characteristics were identified, with 7 ‘internet only’ structural characteristics including embedding, circle jerks, online customer tracking, live remote wagering, multi-lingual sites, increased realism features and remote non-face-to-face medium.
Mills and Nower (2019)	United States	876 adults who had gambled at least monthly in the past year	Assessed the association between trading cryptocurrencies and problem gambling.	Addictive Behaviors (Journal)	Trading cryptocurrencies/high-risk stocks and problem gambling frequency/severity have a strong correlation.
Mishra et al. (2010)	Canada	240 participants from undergraduate psychology classes	Examined whether indicators of risk-propensity, including self-reported personality traits, laboratory-based behavioral measures of risk, and self-reported attitudes toward risk in various domains were associated with general gambling involvement and problem gambling behavior.	Personality and Individual Differences (Journal)	General gambling involvement and problem gambling share common variance with various measures of risk-propensity. There is a positive correlation between risk-taking attitudes and gambling.
Oliveira and Silva (2000)	UK	171 participants surveyed at three gambling settings: bingo clubs, video poker clubs, and a horse-racing club.	Compared sociodemographic attributes, gambling behavior, and the use of drugs between pathological and non-pathological gamblers.	Substance Use & Misuse (Journal)	Stock market investment is deemed to be one of the most “socially acceptable” forms of gambling.
Powell et al. (1999)	Canada	63 students from McGill University	Examined the relationships between risk taking, sensation seeking, and level of gambling involvement.	Substance Use & Misuse (Journal)	Probable/pathological gamblers reported significantly greater risk-taking and sensation-seeking behaviors than those without gambling problems.
Turner (2011)	Canada	1 first person account (David)	Described the personal challenges faced in controlling on-line stock market investing.	Centre for Addiction and Mental Health	Cognitive distortions common to gamblers and traders include selective memory, gambler fallacy, and rationalization.
Wong and Carducci (1991)	United States	233 undergraduate students	Investigated the degree to which the previously established relationship between sensation seeking and risk taking associated with gambling could be extended to everyday financial matters (e.g., personal banking activities).	Journal of Business and Psychology	High sensation seekers displayed greater risk-taking tendencies in everyday financial matters than low sensation seekers.
Youn et al. (2016)	South Korea	1,005 adults who had engaged in financial market investments or trading	Developed a self-rating scale to distinguish gambling addicts in financial markets.	Annals of General Psychiatry (Journal)	This study developed a reliable and valid scale for financial market investments or trading, where a higher score indicates a higher possibility of having a gambling addiction problem in financial markets. Questionnaires used included the DSM-5 diagnostic criteria for gambling disorder and South Oaks Gambling Screen.

#### 3.3.1 Similarities between gambling disorders and excessive trading

Excessive trading and gambling disorders appear to have a positive relationship (Arthur and Delfabbro, 2017; Grall-Bronnec et al., 2017; Marković et al., 2012; Mills and Nower, 2019; Youn et al., 2016). Trading behaviors were found to be associated with increased frequencies, risk, and severity of problem gambling. A study on cryptocurrency trading found that trading and other gambling activities were positively correlated (Mills and Nower, 2019). Gamblers who engaged in trading with both cryptocurrencies and high-risk stocks experienced higher problem gambling than those who engaged in only one form of trading. This has led some financial market investors to seek help for gambling disorders, despite trading being an economic activity for most (Youn et al., 2016). Another study on day trading found that 90.8% of day traders also participated in traditional forms of gambling, which was significantly higher than the general adult population (Arthur and Delfabbro, 2017). This led to a higher prevalence of problem gambling in the day traders as compared to the non-day traders, which could have been attributed to various factors, including increased opportunities for and ease of access to investment and trading (Marković et al., 2012).

Similarly, engagement in gambling behaviors was also associated with increased frequency, risks, and severity of excessive trading. A year-long study found that more than half of regular gamblers were involved in trading cryptocurrency currencies, and those at moderate- and high-risk for problem gambling were more likely to engage in frequent cryptocurrency trading compared to those deemed to be at low or no risk for problem gambling (Mills and Nower, 2019). Notably, those with experience gambling online were observed to have higher rates of cryptocurrency trading compared to those who preferred gambling in physical casinos only. These results can be explained by similarities in the activity profiles of the two behaviors, as well as in the personality profiles and cognitive distortions of those who engage in both activities.

#### 3.3.2 Activity profiles of pathological gambling and excessive trading

The acts of trading and gambling were found to have a considerable overlap in activity profile and structural characteristics (Grall-Bronnec et al., 2017). The view that casino gambling and certain areas of the stock market being essentially one and the same is shared by many, where stock market investment is perceived as a more “socially acceptable” form of gambling (Oliveira and Silva, 2000). Structurally, trading activities also possess the same four components in both traditional and online gambling: “money betting, irreversible betting, a binary win or lose outcome, which depends entirely or partly on chance” (Grall-Bronnec et al., 2017; McCormack and Griffiths, 2013).

Traders and gamblers also demonstrated similarity in the process of developing addictions to their respective activities. Excessive traders initially experienced small wins, following which there was a chasing of losses, and eventually leading to a loss of control. These addictive behaviors identified in the traders allowed them to be characterized as having a gambling disorder (Grall-Bronnec et al., 2017). Of note, traders seemed to have a higher propensity to take part in skill-based forms of gambling as compared to chance-based formats (Arthur and Delfabbro, 2017; Grall-Bronnec et al., 2017).

#### 3.3.3 Personality profiles of pathological gamblers and excessive traders

Studies have found that the personality profiles of some traders and those active in the financial market were similar to those of pathological gamblers (Marković et al., 2012). Sensation-seeking, a personality trait that was common in excessive traders (Grall-Bronnec et al., 2017), had been highlighted by researchers as a key trait in gamblers as well (Golin, 2001; Powell et al., 1999; Wong and Carducci, 1991). Studies have shown that many gamblers partake in such activities for excitement and found similar sentiments in stock traders engaging in trading (Dorn and Sengmueller, 2009; Dorn et al., 2008; Gao and Lin, 2015). Risk-taking was also observed in traders who invested in very high-risk-level stocks with extreme returns (Arthur and Delfabbro, 2017), another personality trait that was found in gamblers as well (Golin, 2001; Powell et al., 1999; Wong and Carducci, 1991). Other studies have found that higher self-reported risk-accepting attitudes were also associated with both excessive stock trading (Markiewicz and Weber, 2013) and excessive gambling (Mishra et al., 2010). Other notable personality traits common to those who participated in gambling and stock investing included having “material needs, competitiveness, superstition, financial conservatism, and numeracy” (Grall-Bronnec et al., 2017; Jadlow and Mowen, 2010).

Persistent loss-chasing, concealing gambling activities, and wagering in response to emotional distress are common behaviors among individuals with gambling disorders (American Psychiatric Association, 2013; Grall-Bronnec et al., 2017). These behaviors are frequently accompanied by underlying psychopathologies, including impulsivity, anxiety, and depression, which may both predispose individuals to wager and be exacerbated by it (Barrault et al., 2017; Dowling et al., 2015).

#### 3.3.4 Cognitive distortions in pathological gambling and excessive trading

Cognitive biases, most significantly the illusion of control, have been observed in both pathological gamblers and excessive traders (Golin, 2001). The illusion of control leads gamblers and traders to think that they have developed strong expertise in their respective fields, resulting in overconfidence and the misconception that all decisions made by them were correct, encouraging them to take unnecessary risks (Grall-Bronnec et al., 2017). Other cognitive distortions found to be common in gamblers and traders included “selective memory, gambler fallacy, and rationalization” (Turner, 2011).

#### 3.3.5 Diagnostic classification for gambling disorders

In the DSM-IV-TR, pathological gambling is described as an impulse control disorder (American Psychiatric Association, 2000) with significant overlaps with psychoactive substance addiction in terms of its biological processes and manifestations of signs and symptoms. Subsequently, in the DSM-V, pathological gambling has since been termed as gambling disorders reclassified under substance-related and addictive disorders (American Psychiatric Association, 2013).

#### 3.3.6 Diagnosability of excessive trading

Many researchers believe that the diagnostic criteria for gambling disorders and addiction can also be applied to excessive trading (Grall-Bronnec et al., 2017; Marković et al., 2012; Youn et al., 2016). Notably, one study developed a new scale, known as the Stock Addiction Inventory, to specifically identify gambling addicts in financial markets, with components adapted from the DSM-V diagnostic criteria (Youn et al., 2016).

The DSM-5 specifies nine criteria for Gambling Disorder, which include: (1) preoccupation with gambling, (2) increasing amounts of money required to achieve excitement (tolerance), (3) repeated unsuccessful attempts to control or stop gambling, (4) restlessness or irritability when attempting to reduce (withdrawal), (5) gambling as a means of escaping problems or alleviating dysphoric mood, (6) chasing losses, (7) lying to conceal involvement, (8) jeopardizing relationships or opportunities, and (2009) relying on others for financial rescue (American Psychiatric Association, 2013). However, these diagnostic criteria are also increasingly acknowledged as applicable to excessive trading behaviors. Research indicates that individuals who engage in excessive trading frequently demonstrate symptoms that are similar to those of gambling disorder, including preoccupation with trading, tolerance, concealment of behavior, and continued engagement despite harm (Grall-Bronnec et al., 2017; Youn et al., 2016; Marković et al., 2012).

When applied to trading, studies have found that majority of the excessive traders met the diagnostic criteria for addictive disorders, including the presence of craving and tolerance behavior, withdrawal symptoms, and negative impact on their daily activities (Marković et al., 2012), as well as gambling disorders, including having a preoccupation with trading, concealment of activities, and inability to control or reduce their trading activity (Grall-Bronnec et al., 2017).

## 4 Discussion

The COVID-19 pandemic’s psychosocial impact likely played a significant role in the exacerbation of both excessive trading and problem gambling behaviors. Economic uncertainty and employment insecurity introduced significant financial pressures, while lockdowns and social distancing measures contributed to increased feelings of isolation, boredom, and stress (Brooks et al., 2020; Islam et al., 2020). These factors may have motivated individuals to pursue emotional escape or a sense of control through speculative financial activities and wagering. Exposure and engagement were further enhanced, notably among younger and psychologically vulnerable populations, as a result of the increase in digital platform usage during this period, which was attributed to increased accessibility and time spent at home (Hodgins and Stevens, 2021; Mills and Nower, 2019).

This narrative review explored the effects of the COVID-19 pandemic on stock and cryptocurrency trading and problem gambling behavior by first examining the relationship between COVID-19 and trading behavior among investors. Next, the effect of COVID-19 on problem gambling activity was summarized. Finally, this review examined the association between trading and problem gambling. We will highlight gaps in the existing literature on trading activity and problem gambling behavior during the COVID-19 pandemic, as well as provide directions for further research.

### 4.1 Effects of COVID-19 pandemic on trading

The effect of the COVID-19 pandemic on investment behavior was multi-fold. This review examined how various factors impacted investor sentiments and psychology during the COVID-19 pandemic. We also examined the effects of the pandemic on trading, including the opening of new brokerage accounts, trading volume, risk tolerance, and herding behavior. The trading behavior adopted by certain groups of investors during the pandemic was worrying as it may potentially mirror the behavior of problem gambling, which is a recognized psychiatric diagnosis.

### 4.2 Future research and recommendations on trading

Particular attention should be paid to investors who are more prone to making risky investment decisions and engage in more trading (i.e., males, younger investors, investors with lower incomes, orphans, and investors with personality traits such as higher neuroticism, lower extraversion, higher openness, higher agreeableness, and lower conscientiousness). Early identification of these high-risk groups can allow family, friends, medical professionals, and the government to intervene before their investment behavior progresses into problem gambling.

As online trading platforms gain popularity among the younger generations of inexperienced traders, this deserves attention from various stakeholders. This is especially since unregulated and risky trading behaviors can eventually evolve into a problem at both a personal and societal level, similar to problem gambling. Future research should follow up on young, inexperienced traders who started investing during the COVID-19 pandemic to determine if they display excessive trading or risky trading behaviors in the long run.

Herding behavior was prominent in various countries and industries during the COVID-19 pandemic. Financial literacy should be promoted, and regulations should be put in place to prevent risky trading behaviors in these vulnerable groups of investors. The government and other relevant authorities could take appropriate steps (for example, by setting regulations and rolling out monetary policies) to reduce uncertainty and mitigate baseless herding behaviors. Further research could be done to follow up on the herding behavior seen in the various markets discussed, to determine if such behavior persists, improves, or worsens post-pandemic.

### 4.3 Effects of COVID-19 pandemic on gambling

The COVID-19 pandemic had not only changed the behavior of investors worldwide but also that of gamblers. The trends in the mode, prevalence, and predisposing factors of gambling behavior since the emergence of the COVID-19 pandemic were explored. New restrictions in movement have changed the way people participate in gambling activities, with gamblers shifting their betting activities from physical venues to online platforms. While online gambling itself may not predispose individuals to gambling addiction disorder, the use of internet gambling among highly engaged gamblers can contribute to gambling problems. Hence, further research of this mode of gambling can be useful to inform the development of preventative measures and better regulation of online gambling activities.

This paper also identified a heterogeneous response in individual gambling behavior, with fewer people participating in gambling activities in general, but a specific minority exhibiting potentially problematic gambling behaviors. Factors associated with greater problematic gambling activities had been explored, such as being in the 18–25-year-old age group, along with psychological factors such as loneliness, depression, and anxiety, and motivations for stress relief and boredom. With more young adults at risk of problematic gambling during the COVID-19 pandemic, strategies to address this issue could adapt to target this demographic group. Future research can focus on evaluating psychological and behavioral treatments specifically for individuals who participate in gambling due to psychological reasons, as well as to identify other relevant risk factors for pathological gambling amidst the pandemic.

Of note, those who increase their gambling activities during the COVID-19 period may be more susceptible to gambling disorders in the future. One study suggested that 48% of people who increased their gambling during the lockdown period either maintained or further increased such activities after the relaxation of restrictions. Further studies can explore if there is an association between gambling activities during financial uncertainty and subsequent gambling behavior beyond times of crisis.

### 4.4 Harmful consequences of excessive trading and gambling disorders

Excessive trading and pathological gambling can be seen as two sides of the same coin.

Traders that engage in excessive trading often fulfill the criteria for gambling disorders in the DSM; likewise, those at risk of problem gambling are also more likely to engage in excessive trading. Both groups share several similarities in personality traits and cognitive distortions, as well as other characteristics.

This is significant given the possible negative sequelae arising from the two activities. Excessive trading is associated with comorbid psychiatric disorders such as depression and anxiety. Gamblers are also at risk of these psychiatric disorders, with three-quarters of the patients in a study suffering from major depression and a quarter from anxiety. This is supported by other studies which found that comorbid psychiatric disorders were common in those with gambling disorders. Gamblers who took part in trading of both cryptocurrency and high-risk stocks were shown to have greater depression and anxiety symptoms than those who took part in trading of either cryptocurrency or high-risk stocks, suggesting that an increase in the volume of trading is significant in predicting a greater risk of having these comorbidities. Apart from psychiatric disorders, gambling disorders and excessive trading can lead to a loss in financial and psychosocial functioning and were also found to be associated with substance abuse.

### 4.5 Limitations

One limitation of this narrative review is its susceptibility to selection and reporting biases, as the data were exclusively drawn from published studies, potentially reflecting selective perspectives while omitting unpublished or non-English sources. Additionally, this review does not fully account for regional variations in the impact of the COVID-19 pandemic on trading and gambling behaviors. While our analysis highlights significant shifts in gambling patterns during the pandemic, we acknowledge that differences in lockdown measures across regions may have allowed offline gambling to persist in certain areas. Moreover, the specific effects of the pandemic on individuals with pre-existing gambling problems, as well as those who had never engaged in gambling before, remain underexplored. Future research should aim to provide a more comprehensive understanding of these dynamics to better inform public health interventions and regulatory frameworks.

### 4.6 Recommendations

The robust interplay between excessive trading and problem gambling during the COVID-19 pandemic can lead to compounding of the downstream effects that may continue to linger on in the generations to come. This is a cause for concern given the rising prevalence of these activities in the community. Thus, future research should be directed toward early identification of excessive traders, given their increased risk of problem gambling. Future work should also explore if current treatments for pathological gamblers or gambling disorders can be adapted for use in excessive traders, given the similarities in the activities and traits of people who engage in them. It is evident that any future research that seeks to expand upon the results of this analysis should concentrate on the psychological and behavioral causes of excessive or problematic gambling. It has been suggested that the online environment presents a greater risk than other settings, necessitating the implementation of prevention measures that are specifically designed for this environment. Incorporating built-in mechanisms to restrict excessive spending on the platforms and increasing public awareness of the dangers associated with these online platforms are potential solutions. Additionally, authorities may contemplate the approval of harm reduction strategies that are specifically designed for vulnerable populations, such as novice or younger users. It is reasonable to provide recommendations and address these concerns in a more effective manner in order to achieve the objectives outlined in clinical and social contexts.

## 5 Conclusion

In conclusion, this narrative review sought to explore how excessive trading and risky trading behaviors have become more prominent during the early phase of the COVID-19 pandemic. Our findings were concerning given that excessive trading shares many similar characteristics and implications as problem gambling – which is a well-recognized psychiatric disorder. We propose that excessive trading could indeed be a form of addiction, akin to a type of gambling disorder, which could be potentiated by the changes in the way we live during the early COVID-19 pandemic. Given the potential negative consequences of excessive trading for individuals (including functional impairment) and larger communities, excessive trading behavior deserves greater attention from the psychiatric community and governments around the world, such that appropriate regulations can be put in place and relevant aid can be promptly provided by relevant authorities.
